# Random and Directed Walk-Based Top-*k* Queries in Wireless Sensor Networks

**DOI:** 10.3390/s150612273

**Published:** 2015-05-26

**Authors:** Jun-Song Fu, Yun Liu

**Affiliations:** School of Electronic and Information Engineering, Key Laboratory of Communication and Information Systems, Beijing Municipal Commission of Education, Beijing Jiaotong University, Beijing 100044, China; E-Mail: 14111005@bjtu.edu.cn

**Keywords:** wireless sensor networks, top-*k* query, random walk, directed walk, energy efficiency

## Abstract

In wireless sensor networks, filter-based top-*k* query approaches are the state-of-the-art solutions and have been extensively researched in the literature, however, they are very sensitive to the network parameters, including the size of the network, dynamics of the sensors’ readings and declines in the overall range of all the readings. In this work, a random walk-based top-*k* query approach called RWTQ and a directed walk-based top-*k* query approach called DWTQ are proposed. At the beginning of a top-*k* query, one or several tokens are sent to the specific node(s) in the network by the base station. Then, each token walks in the network independently to record and process the readings in a random or directed way. A strategy of choosing the “right” way in DWTQ is carefully designed for the token(s) to arrive at the high-value regions as soon as possible. When designing the walking strategy for DWTQ, the spatial correlations of the readings are also considered. Theoretical analysis and simulation results indicate that RWTQ and DWTQ both are very robust against these parameters discussed previously. In addition, DWTQ outperforms TAG, FILA and EXTOK in transmission cost, energy consumption and network lifetime.

## 1. Introduction

Wireless sensor networks (WSNs) composed of a large number of wireless connected devices have been widely researched and applied in many fields. Oftentimes, there is a powerful base station (BS) acting as a bridge between the WSN and the external users. On the contrary, the other nodes have very limited resources to collect, process, transmit and receive the data about the surrounding physical environment making energy conservation is a major issue. Top-k queries, *i.e*., querying the top-k readings with the corresponding nodes of a WSN, is a very common demand for the users and has been widely studied in the literature [1,2,3,4,5,6]. The top-k query approaches can be roughly divided into two categories, *i.e*., aggregation-based and filter-based query approaches. There are some parameters of the networks and the readings such as large size, severe damages to the network topology, and dynamics of the nodes’ readings that can affect the performance of the query approaches. Aggregation-based query approaches perform well in defending against these parameters. In fact, for most of aggregation-based query approaches, the transmission cost is stable for a specific query. In most cases, the filter-based top-k query approaches outperforms aggregation-based top-k query methods in transmission cost, energy consumption and network lifetime. However, the updating of the filters is very sensitive to the parameters discussed previously, especially the size of the network and the dynamics of the readings, which will be discussed in detail in Section 5.1 based on a simple model.

Another very important parameter that influences the performance of the filter-based top-k query approaches is the decline of the whole range of readings. Theoretical analysis in Section 5.1 illustrates that the declining speed of the whole readings range has a significant bad impact on the performance of filter-based top-k query approaches. The filters easily become heavy burdens rather than something that reduces the transmission cost when all the readings decline. In real life, the decline of the readings is a very common phenomenon.

Consider a WSN deployed in an interest region which is used to monitor the temperature and the user wants to know the top-k readings in the network. From morning to afternoon, the trends of all the temperatures are increasing though some exceptional cases could happen because of measurement error or failure of the nodes. In this period, the filter-based top-k query approaches outperform the approaches based on aggregation techniques. However, the whole range of temperatures decreases from afternoon to dawn. In this condition, the filters would change to be the heavy burden of the network.

Power supply is strictly limited in WSNs and therefore residual energy of the nodes is an essential network management parameter. Therefore, the users may monitor the top-k nodes with the highest residual energy. Obviously, the residual energy for a node always decreases during the whole lifetime which can lead to the failure of the filter-based approaches.

Another shortcoming of filter-based top-k query approaches is that though the filter-based approaches carefully consider the temporal correlations of the readings, the spatial correlations between them are ignored, but spatial correlations in fact are very common and have been confirmed by analysis of real data set in various papers [7,8,9].

Consider a WSN which is designed to monitor gas concentrations in factories. When there is a gas leakage incident, the gas will diffuse from the source to the surrounding environment. Under normal conditions, the readings of the nodes near the gas source will be beyond the safety threshold, however the remote nodes will not detect the incident. Then it is likely that the nodes that detect the gas coordinate with their neighbors rather than the remote ones to verify the incident.

The distribution of the transmission cost is an important indicator of the load-balance of a WSN. Therefore, the users may be interested in finding the nodes with top-k traffic. When a node’s transmission cost is very high, we would draw a conclusion that the transmission costs of the node’s neighbors are also very likely to be high. This is reasonable because, in most cases, a node just communicates with its neighbors rather than nodes in a remote region. As a result, the neighbors of the node have a high probability of having a high transmission cost.

In fact, the strong relations between nodes with their neighbors are the basis of many in-network data processing techniques, such as data compression, data prediction and data fusion in WSNs. In addition, the relations can also be used in top-k queries. In this work, we present an in-depth analysis on filter-based top-k query approaches and propose two novel top-k query methods named RWTQ and DWTQ to overcome the shortcomings of filter-based top-k query approaches. In RWTQ, each node uses its Relative-Neighbors’ list only to make the token(s) walking decisions. We employ the Relative-Neighbors of a node rather than the all the neighbors to reduce the redundant paths.

On the contrary, DWTQ uses not only the position of the node but also the *a priori* information stored in the token to make the token(s) walking decisions. DWTQ is an extension of RWTQ and comprises of four modes, *i.e*., Random-Walk (RW) Mode, Directed-Walk (DW) Mode, Extreme-Point (EP) Mode and Leave (L) mode. A token can switch mode between these four modes. The contributions of this paper are summarized as follows:

(1) We find and point out the limitations of filter-based top-k query approaches in certain situations based on theoretical analysis presented in Section 5.1. We employ a simple network model which is composed of N nodes and make locate the base station at the center. The relations between the performance and the parameters of filter-based top-k query approaches are analyzed in detail. Analysis results are shown with figures.
(2) A novel paradigm called RWTQ is proposed. The whole framework of RWTQ is displayed, which is also the basis of DWTQ. In RWTQ, we introduce the Relative Neighborhood Graph (RNG) to defend against the Density-Trap phenomenon. A distributed construction method for RNG is also discussed.
(3) We extend the RWTQ to DWTQ considering the spatial correlations between the readings of the nodes. DWTQ comprises four modes, *i.e*., Random-Walk (RW) Mode, Directed-Walk (DW) Mode, Extreme-Point (EP) Mode and Leave (L) mode. We provide a detailed discussion of each type of mode and the switches between the modes.
(4) We evaluate the performance of RWTQ and DWTQ through a series of simulations. The results show that DWTQ outperforms RWTQ, TAG, FILA and EXTOK in transmission cost, energy consumption and network lifetime.

The rest of this paper is organized as follows: Section 2 reviews the related work on top-k query approaches. Section 3 gives the background of top-k queries and our random walk-based approach, RWTQ, in detail. We then extend the RWTQ to the directed walk-based top-k query approach, DWTQ, and design a detailed strategy of walking directions in Section 4. Both theoretical analysis and simulation are employed to evaluate the performances of RWTQ, DWTQ and some other approaches in Section 5. Finally, the conclusions of the paper are presented in Section 6.

## 2. Related Work

As discussed previously, the top-k query problem in WSNs has been widely studied and most of the previous approaches are divided into two categories, *i.e*., aggregation-based and filter-based top-k query approaches, and we present them, respectively, in the following paragraphs.

Several data aggregation functions exist in the literature, including sum, count, average, min, max and so on, and the top-k query problem is just one special case of them. As a result, most data aggregation researchers focus on constructing the routing architecture and reducing the transmission cost. TAG [3] is a well-known aggregation algorithm which can be used to solve the top-k query problem. Any routing algorithm can be used by TAG for communications between the base station and all the nodes in a network. A series of aggregation functions (e.g., MAX, MIN, they are also capable of querying the top-k readings. In [10], a clustered aggregation approach (CAG) is presented which outperforms TAG in transmission cost. The disadvantage of CAG is that the hot-spot nodes are more easily exhausted, which shortens the lifetime of networks significantly. There are many other protocols that can be used to aggregate the data such as LEACH [11], directed diffusion [12] and GPSR [13].

Range caching [4], proposed by Olston *et al*., is the rudiment of filter-based top-k queries. In range caching, the data cache stores an interval approximation which is a value range for each data source. When the data value of a source changes, it would be transmitted to the data cache only when the value is beyond the interval approximation. Therefore, the transmission cost is reduced when the precision (width of the interval approximation) is set appropriately. A parameterized algorithm for adjusting the precision of approximations is designed to get the best performance as data value, precision or workload vary. Then, in [5], Babcock and Olston extended the approach in [4] and applied it to the top-k monitoring problem in data streams. Initially, the coordinator node computes and maintains a top-k set and installs arithmetic constrains at each monitor node. For each monitor node, if the updated value is located in the arithmetic constraints, no information needs to be transmitted to the coordinator node, which can reduce the communication cost. When some constraint is violated, a process called resolution takes place which can determine whether it is necessary to impose new constraints on the monitor nodes. To our knowledge, Olston *et al*. first proposed in [6] the use of adaptive filters to continuously query over distributed data streams with low communication overhead. They designed a low-overhead algorithm for setting the widths of the filters adaptively which always guarantees precision constrains of the users will be met. FILA [1] is another classic query algorithm which uses range-based filters to reduce the transmission cost and save energy. In addition, it is developed specifically for WSNs. Each sensor node installs a filter locally and, for the top-k members, the filter is unique in the whole network; all the other nodes share a same filter. The sensor-initiated updates are divided into three types: Internal update, Join update and Leave update. For each type of updates, a corresponding mechanism is used to reinstall the filters. When the updated readings of the sensor nodes do not surpass the filter, it has no need to transfer the readings to the base station (BS). More recently, a new filter-based top-k query approach called EXTOK [2] was developed. Different from FILA, all the nodes in EXTOK shares the same filter which is a number rather than a range and the top-k nodes always upload the readings to the BS. On the contrary, the other nodes upload the readings only when the value of their readings is larger than the filter. Different from the above two categories of traditional top-k query approaches, in this work we propose a novel method based on the walking of the token in the network to collect the top-k readings. To our knowledge, this is a new perspective on the top-k query problem.

## 3. Top-*k* Query Based on Random Walk

In Section 3.1, we first state the problem of top-k queries in WSNs and then present some assumptions to make our approaches work well. RWTQ is discussed in Section 3.2.

### 3.1. Problem Definition and Assumptions

Considering a monitoring region in which a large number of homogeneous nodes are deployed randomly, we assume that all the nodes are static and are capable of collecting information, processing, transmitting and receiving data. In addition, each node is assumed to know its own geographic position either from a GPS device or by some other means. Every node measures the local physical phenomenon (e.g., temperature, humidity, residual energy and concentration of toxic gases) with a constant sampling rate. In each sampling period, the top-k readings and the corresponding nodes in the whole network are required by the external users. A more formal definition of the top-k query problem is given as follows:

Given a network which comprises a set of nodes $Nodes=(n_{1},\;n_{2},\ldots ,n_{N})$, all the nodes generate local readings $R=\left(R_{n_{2}},R_{n_{2}},\ldots ,R_{n_{N}}\right)$ synchronously with a constant frequency. In each period, the users want to get a list L containing k pieces of records shown as follows:

(1) $$L=<\left(n_{1}^{'},R_{n_{1}^{'}}\right),\left(n_{2}^{'},R_{n_{2}^{'}}\right),\ldots ,\left(n_{k}^{'},R_{n_{k}^{'}}\right)>$$

where $R_{i}^{'}$ is the reading of $n_{i}^{'}$:

(2) $$\forall 0<i<j\leq k,\;\;R_{n_{i}^{'}}\geq R_{n_{j}^{'}}$$

and:

(3) $$\forall n_{l}\in Nodes\;\text{and}\;n_{l}\notin n_{i}^{'}\;\left(i=1,2,\ldots ,k\right),\;R_{n_{l}^{'}}\leq R_{n_{k}^{'}}$$

For ease of description, in this work, we see the set of homogeneous nodes with identical circular communication range r as a graph G. The vertex of the graph G comprises all the nodes in the network. If the distance $d\left(s_{i},\;s_{j}\right)$ between two nodes $n_{i}$ and $n_{j}$ is smaller than r, the two nodes can communicate with each other and an edge exists between $n_{i}$ and $n_{j}$ in G. It is easy to find that G is an unweighted and undirected graph. We can get a distributed G by each node communicating with its neighbors and a full list L of a node’s neighbors can be obtained by each node. For this, it is essential to distinguish individual neighbors. Any locally unique identifier can be used for this propose, e.g., unique IDs in the network, 802.11 MAC addresses [14] or Bluetooth cluster addresses [15]. In this work, we assume that all the nodes are static and the topology is stable. Therefore, the neighbors list L can be updated by the nodes with a long time interval.

### 3.2. RWTQ

At the beginning of the network construction, each node sends information about its location to the BS. To avoid sending some of the tokens to the neighboring nodes, the BS selects n representative nodes in the network based on an algorithm named SelRep, where n is the number of tokens preset by the users, specified by the users. The pseudo-code of SelRep is shown as follows:
**Algorithm 1:**
SelRep**Input:** locations of all the nodes Locations and parameter n**Output:**
n representations1) **while** the number of clusters > n2)    combine the nearest two clusters3) **end while**4) **for** each cluster5)   select a representation of the cluster6) **end for**

Having obtained n representations, BS sends a token to each of them by any routing algorithm and then the tokens walk in the network randomly to collect the top-k readings. In this work, we employ GPSR [13] to exchange data between BS and the nodes, because GPSR has strong correlations with our approaches (as an example, they both employ the *Relative Neighborhood Graph* which will be introduced in the following section). Each token has a unique ID and a pedometer which is initiated by the representations.

A node that receives a token first checks the pedometer and if the pedometer is smaller than a threshold T, adds one to the pedometer count. Then, the node needs to search the readings cache for a matching reading. Note that, the readings in the token are sorted in descending order and the node compares the local reading with the readings in the token in order. When finding a smaller reading in the token, the node inserts the local reading before the smaller reading. Then, the node needs to check the number of the readings in the token, if the number beyond k, deletes the last reading in the token; else, does nothing. Having updated the token, the node chooses one neighbor from the neighbor list L (excluding the neighbor that sends the token) with equal probability and sends the token to the neighbor. The pseudo-code of the Updating-Token algorithm is as follows:
**Algorithm 2:** Updating-Token**Input:** A token and its readings sorted in descending order**Output:** An updated token1) **for**
*i*=1 to the number of the readings in the token2)   scan the readings in order3)   **if** (the local reading > the *i*-th reading in the token)4)    insert the local reading before the *i*-th reading5)    **break**6)   **end if**7) **end for**8) **if** the number of readings in the token beyond *k*9)  delete the last reading in the token10) **end if**

**Figure 1:**
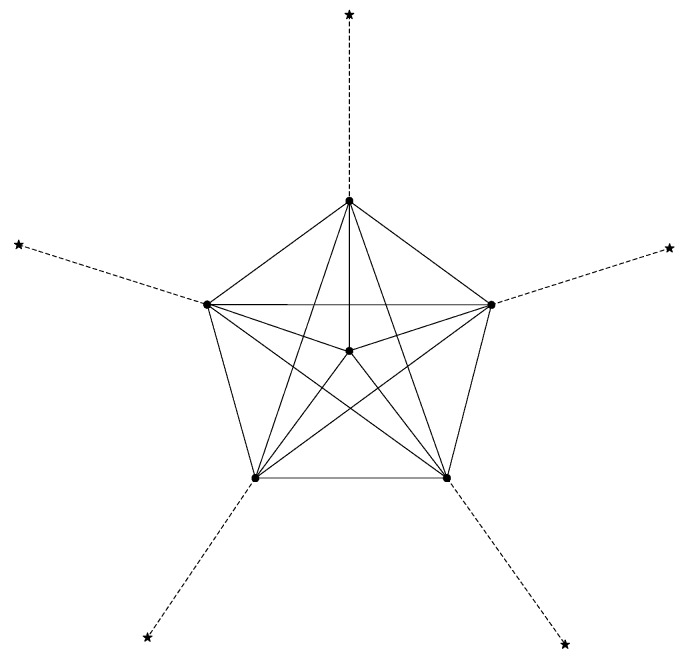
The token trapped by a high-density region.

On the contrary, when a node finds that the pedometer is beyond a threshold T, it realizes that the token should be sent to the BS by GPSR and then selects the next hop with the rules in [13]. Using the full list of neighbors to decide the next hop comes with one attendant drawback named Density-Trap (D-T): it is most likely that in a high-density region (H-R) the token will walks around and around, and it is hard to walk out. A simple example of such situation is shown in Figure 1. Here, the six black dots comprise an H-R and they can communicate with each other directly, *i.e*., each pair of them are neighbors. In addition, there are five stars and each star connects with the H-R by a “narrow bridge”, *i.e*., each star can only communicate with one black dot. Considering that a token randomly walks in the H-R, in each step, the probability that the token is sent to the stars is smaller than 1/5, because for a black dot located at the border of H-R, it sends the token to a star with a probability of 1/5, for the black dot locating at the center, it can’t send the token to the stars. As the H-R’s density increases, the probability that a token will walk out of H-R decreases, which would consume lots of energy and does not help get the top-k readings in the network.

**Figure 2:**
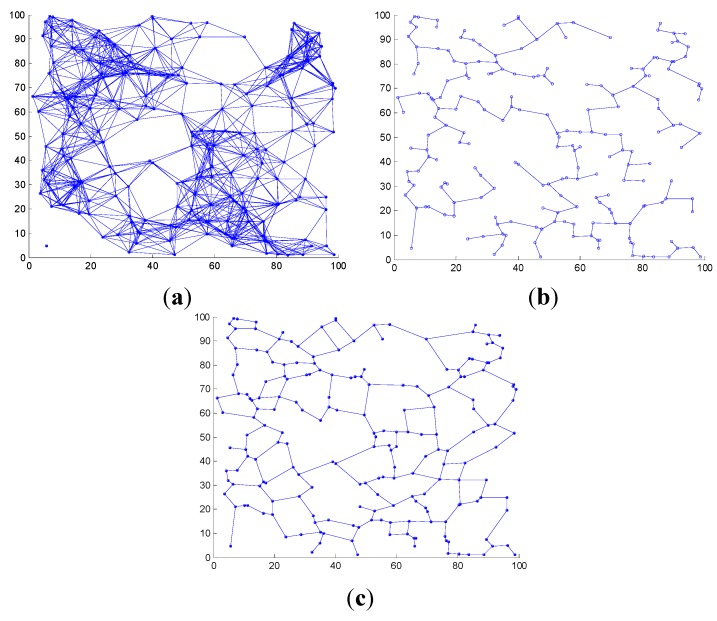
Comparison of (**a**) The full graph G; (**b**) MST of G; (**c**) RNG of G.

Motivated by the D-T problem, we note that the full graph G, shown in Figure 2a, is not suitable for a token to randomly walk on, because there are some redundant choices, especially for an H-R when choosing the next step for a token. An intuitive choice is to let the tokens walk on the Minimum Spanning Tree (MST) of G, as shown in Figure 2b, which can solve the D-T problem. However, walking on MST, the tokens always walk to the dead end and as a result, the tokens have to go back along the way they walked. Therefore, we employ the Relative Neighborhood Graph (RNG) [16], as shown in Figure 2c, which is a well-known planar graph to solve the D-T phenomenon. RNG is a subset of the full graph G and a superset of MST. In Figure 2, a comparison of the full graph G, its MST and RNG are presented.

As in [13], given a collection of vertexes C with known locations, the Relative-Neighbors (RNs) and the RNG are defined as follows:

Given two points $v_{i}$ and $v_{j}$ in C, they are RNs if, for each $v_{k}\in C$, $d\left(v_{i},\;v_{j}\right)\leq \text{max}\left[d\left(v_{i},\;v_{k}\right),d\left(v_{j},\;v_{k}\right)\right]$.

An edge in RNG exists between vertexes $v_{i}$ and $v_{j}$ for all i, j=1,2,…,N, i≠ j, if and only if the two vertexes are RNs.

Several algorithms have been proposed for constructing RNG [17]*.* To reduce the transmission cost in the network, we employ a distributed fashion algorithm proposed in [13]. Given the full list of the neighbors L, each node can get the RNs-List RNs−L as follows:
**Algorithm 3:** Constructing RNG**Input:** Full list of the neighbors, L**Output:**
RNs−L of the node1) **for** each $v_{i}\epsilon L$2)  **for** each $v_{j}\epsilon L$3)   **if**
$v_{i}==v_{j}$4)    **continue**5)   **else if**
$d\left(v_{i},\;v_{j}\right)>\text{max}\left[d\left(v_{i},\;v_{k}\right),d\left(v_{j},\;v_{k}\right)\right]$6)    eliminate edge $\left(v_{i},\;v_{j}\right)$7)    **break**8)   **end if**9)  **end for**10) **end for**

Based on the pseudo-code shown in Algorithm 3, each sensor node can get the RNs−L. As a result, each sensor node can send the tokens based on the RNs−L rather than L which can significantly release the D-T problem. In addition, employing the RNG makes it easy to use GPSR to exchange data between BS and the nodes.

## 4. Extending Random Walk to Directed Walk

In Section 3, we developed a top-k query approach based on a random walk, which is suitable for WSNs in which each node’s readings are absolutely independent of any other nodes’ readings. However, as described in Section 1, the reading of a node has strong correlations with that of its neighbors, because the information of most physical phenomena strongly correlates to spatial locations. To further improve the efficiency and reduce transmission cost, we propose the aggressive use of spatial correlations. RWTQ is thus extended to DWTQ which carefully considers these spatial correlations.

**Figure 3:**
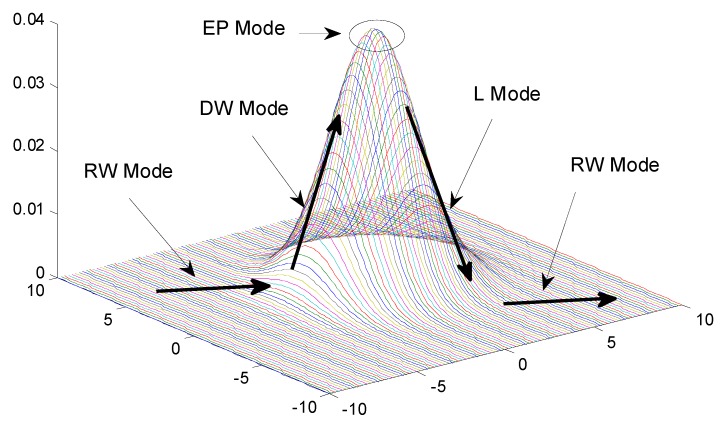
The “mountain” the data and DWTQ algorithm.

As shown in Figure 3, there is a “mountain” with an extreme point and DWTQ is comprised of four modes, *i.e*., Random-Walk (RW) Mode, Directed-Walk (DW) Mode, Extreme-Point (EP) Mode and Leave (L) mode, to search the extreme point efficiently. Initially, there is no information about which direction the token should walk and then get the top-k readings with a high probability. Therefore, the token needs to collect and process the information of the readings by RW Mode which is slightly different to RWTQ. When a node finds that there is a clear target direction in which the values of the readings always increase, the mode of the token is changed to DW Mode until the value of the readings reach an extreme point where the mode of the token is changed to EP Mode. After EP Mode, the token’s mode becomes L Mode immediately, which can lead the token out of the “mountain” quickly and then becomes RW Mode when the node finds that the value of the readings stops decreasing. If the pedometer count is smaller than a threshold, the mode of the token can switch between these four modes; if the pedometer count is larger than a threshold and the mode of the token is not DW and EP Mode, the token is transmitted to the base station directly; if the pedometer count is larger than a threshold and the mode of the token is DW Mode, the token is transmitted to the base station after the mode of the token changes to L Mode.

The four modes, *i.e*., RW, DW, EP, and L mode, are presented in Section 4.1, Section 4.2, Section 4.3 and Section 4.4, respectively.

### 4.1. RW Mode

The only different point between RW Mode and RWTQ is that the token has to record the information which would be used to decide its walking direction. In this work, the token records the latest l readings and their locations, called Discover-Information, in RW Mode. The nodes receive a RW-Mode token need to analyze the Discover-Information to check whether there is a clear target direction in which the readings always increase.

**Figure 4:**
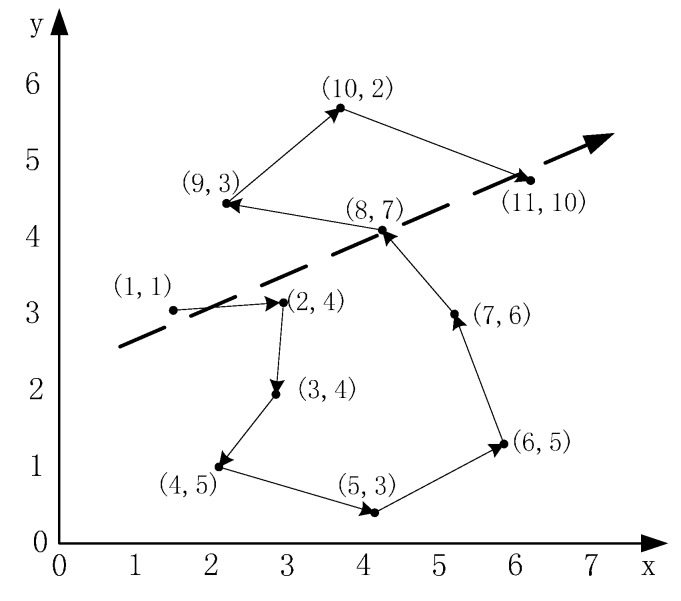
The route of a RW Mode token.

In the example shown in Figure 4, there are 10 records stored in the token and a record which is generated by the node itself. For each record, the first part in the braces is the order number and the second part is the reading value. In Figure 4, there is a clear target direction presented by the arrow in which the readings always increase and, intuitively, the token should walk down in the arrow’s direction. We design the Decide-Direction algorithm to find the clear target direction. Assume that l pieces of records are contained in a token of the form [${\text{Reading}}_{i},{\text{Location}}_{i}$] as shown in Table 1, where i=1, 2, …, l.

**Table 1:** The Discover-Information contained in the token.

Order	Reading	Location (*x*, *y*)
1	1	(1.5, 3.0)
2	4	(3.0, 3.3)
3	4	(2.8, 1.9)
4	5	(2.3, 0.9)
5	3	(4.3, 0.4)
6	5	(5.8, 1.3)
7	6	(5.4, 2.9)
8	7	(4.3, 4.0)
9	3	(2.2, 4.4)
10	2	(3.7, 5.7)
11	10	(6.3, 4.7)

**Figure 5:**
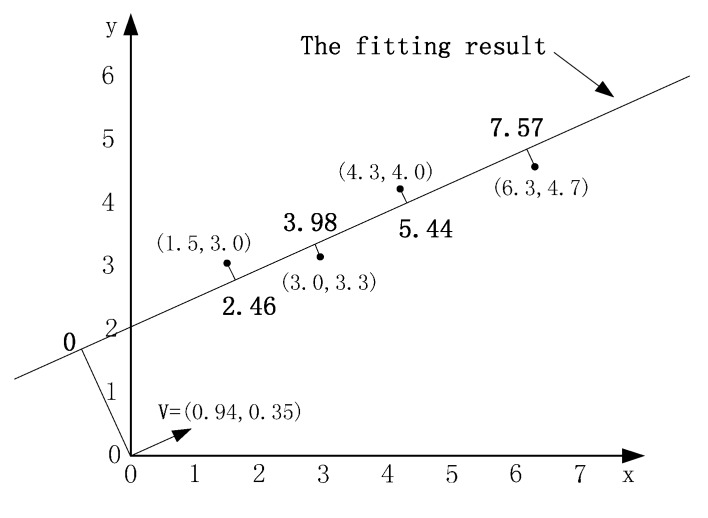
An example of fitting the locations.

An important parameter is n which indicates the number of the nodes that comprise the arrow. The larger of n, the more accurate the target direction. In order to get the arrow, there must be n nodes nearly located on a line which can be indicated by |Corr(X,Y)|, where X and Y are the sets of *x* coordination and *y* coordination values of the n nodes. Corr(X,Y) can be calculated as follows:

(4) $$\text{Corr}\left(X,Y\right)=\frac{\text{Cov}\left(X,Y\right)}{\sqrt{\text{Var}\left(X\right)}\sqrt{\text{Var}\left(Y\right)}}$$

where Cov(X,Y)=E[(X−E(X))(Y−E(Y))]. As examples, the Corr(X,Y) of 1st, 2nd, 8th and 11th records is 0.9888 and that of 6th, 7th, 8th and 10th is −0.9710. If the absolute value of Corr(X,Y) beyond a threshold t, we need to fit the locations of these records by the least square methods and get the direction vector v; as shown in Figure 5. Then, a location (x,y) can be mapped to a one-dimensional point xy locates on the fitting result by Equation (5):

(5) xy=(x,y)⋅v

As shown in Figure 5, the locations (1.5, 3.0), (3.0, 3.3), (4.3, 4.0), (6.3, 4.7) can be reduced to the responding values 2.46, 3.98, 5.44, 7.57, respectively. Based on the responding value, the locations can be sorted in ascending order, *i.e*., (1.5, 3.0) < (3.0, 3.3) < (4.3, 4.0) < (6.3, 4.7). If the readings always increase with the sorted locations, the direction of *v* is where the token should walk down; else if the readings always decrease with the sorted locations, the negative direction is where the token should walk down; otherwise, there is no target direction.

Note that, to reduce time complexity of Decide-Direction algorithm, the node has no need to consider all the combinations of n locations and can find all the sets of n−1 locations and add its own location to them to comprise n locations. This is reasonable, because the previous node has checked most of the combinations of n locations. Obviously, if there is no clear target direction, the token continues walking in the RNG of the full graph.

The pseudo-code of Decide-Direction algorithm is shown as follows:
**Algorithm 4:** Decide-Direction**Input:** Discover-Information, *i.e*., l pieces of records**Output:** The direction vector1) **for** each n locations2)  **if** the covariance coefficient of X and Y don’t beyond *t*3)   break4)  **else**5)   fit the locations by least square method and get v6)   map the locations to a one-dimensional value locating on the direction of fitting result7)   sort the locations by the value in ascent order8)   **if** the readings always increases with the sorted locations9)   the direction of v is target direction10)   **else if** the readings always decreases with the sorted locations11)   the negative direction of v is target direction12)  **else**13)   there is no clear target direction14)  **end if**15)   **end if**16) **end for**

### 4.2. DW Mode

When a node receives a RW Mode token and finds that there is a clear target direction, it will first change the token’s mode to DW Mode. Because the direction of v is decided by n pieces of records rather than all the records contained in the token, the redundant records can be deleted. A node that receives a DW Mode token needs to fine tune the target direction based on its own locations and the method is the same to algorithm of Decide-Direction. A big challenge for a node is to decide which neighbor is the best choice to send the token. To get the best result, each node sends the token to one node in the full list of neighbors rather than in that of the RNG-Neighbor and before sending the token, it needs to collect the readings of its neighbors that locate “close” to the direction of v and has a reading with high value.

In this work, when node i choices its neighbor, node j that “close” to the direction of v means that the included angle between v and i→j is smaller than a threshold ang. If several nodes are all “close” to v, the node k with the highest reading $R_{k}$ is chosen as the next hop of the token if $R_{k}\geq R_{i}$. If there is no node with a reading higher than $R_{i}$ that close to v, send the token to the node in the full list of neighbors with highest reading $R_{m}$, if $R_{m}\geq R_{i}$. However, if all the neighbors of i have no readings higher than $R_{i}$, node i changes the token’s mode to EP Mode.

### 4.3. EP Mode

When a node receives a token with DW Mode and finds that all the readings of its neighbors are smaller than its own reading, the token will be switched to EP Mode. In EP Mode, the node needs to collect all the readings of its neighbors and update the token based on the readings which is presented in Figure 2. After EP Mode, the token’s mode is switched into L mode immediately.

### 4.4. L Mode

When a node receives a token with L mode; it realizes that the token should be transmitted to the region that out of the “mountain” of the data. L mode is a reverse mode of DW Mode. The only difference between L mode and DW Mode is that the token walks in the negative direction of the target direction in Section 4.1. When a node finds that there is no neighbor close to v that has a smaller reading than itself; the token is out of the mountain region and the token’s mode will be switched into RW Mode.

As discussed previously, the most important content in this section is how to decide the direction that the token(s) walk down, therefore we called the method DWTQ. In the next section, we evaluate the performance of RWTQ and DWTQ, and compare them with the aggregation-based top-k query approach TAG, filter-based top-k query approaches FILA and EXTOK in transmission cost, query accuracy, energy cost and network lifetime.

**Table 2:** Parameters and their meanings.

Domain of the Parameters	Symbol	Meaning
Query Model	*k*	Number of readings queried by the BS
Network Topology	*n*	Number of nodes in each row
	*N*	Total number of nodes in the network
	*d*	Distance between neighboring nodes
Message Model	$l_{id}$	The size of a sensor identify
	$l_{r}$	The size of a sensor reading
	$l_{fk}$	The size of a filtering window for the top-*k* readings
	$l_{fn}$	The size of a filtering window for the non-top-*k* readings
	$l_{p}$	The size of a probe message
Energy Cost Model	α	Electronics energy
	β	Amplifier energy
	q	Attenuation coefficient
Dynamics of the Readings	$R_{i}$	The *i*-th reading of the sensors in descending order
	$f_{i}$	The filter between the *i*-th and (i+1)-th reading
	*w*	The average window width of the readings
Physical Phenomenon Model	$P_{max}$	The maximum value of physical phenomenon
	$P_{min}$	The minimum value of physical phenomenon
	$P_{i}$	The *i*-th real value of physical phenomenon in descending order
	$M_{i-phy}$	The mean value of the *i*-th physical phenomenon
	$Var_{i-phy}$	The variance of the *i*-th physical phenomenon
Measurement Error Model	$E_{i}$	The measurement error of *i*-th reading
	$M_{i-err}$	The mean value of the *i*-th measurement error
	$Var_{i-err}$	The variance of the *i*-th measurement error

## 5. Theoretical Analysis and Simulation

In this section, we evaluate the performance of RWTQ and DWTQ by both theoretical analysis and simulation. First, in Section 5.1, we discuss how the performances of the filter-based approaches are affected by the size of the network, dynamics of sensors’ readings and decline of the whole readings’ range through a theoretical analysis based on a simple model. We first set up a simple wireless senor network with a square topology and then model the message, energy consumption, readings, physical phenomenon and the measurement error. The performance of filter-based approaches is compared with that of a representative aggregation-based approach TAG [3]. The analysis results are presented in Figure 8, Figure 9, Figure 10 and Figure 11. Through theoretical analysis, we can find that the filter-based approaches are useless in certain situations and it is essential to develop a novel top-k query method. Then, in Section 5.2, Section 5.3, Section 5.4 and Section 5.5, we use the simulator ns-3 [18] (version 3.21) to evaluate the performances of RWTQ and DWTQ. We compare them to TAG, FILA and EXTOK in terms of transmission cost, query accuracy, energy cost and network lifetime. Finally, in Section 5.6, we give a concluding discussion of the simulations. Table 2 is given for users to index the parameters.

### 5.1. The Failure of Filter-Based Top-*k* Query Approaches

Various metrics can be employed to evaluate the performance of a top-k query approach and transmission cost is one of the most essential metrics. Therefore, our goal is to analyze the average transmission cost of filter-based approaches with different sizes of a network and the dynamics of the readings. The transmission cost is defined as the total amount of data transmitted in the whole network in a round of a top-k query.

**Figure 6:**
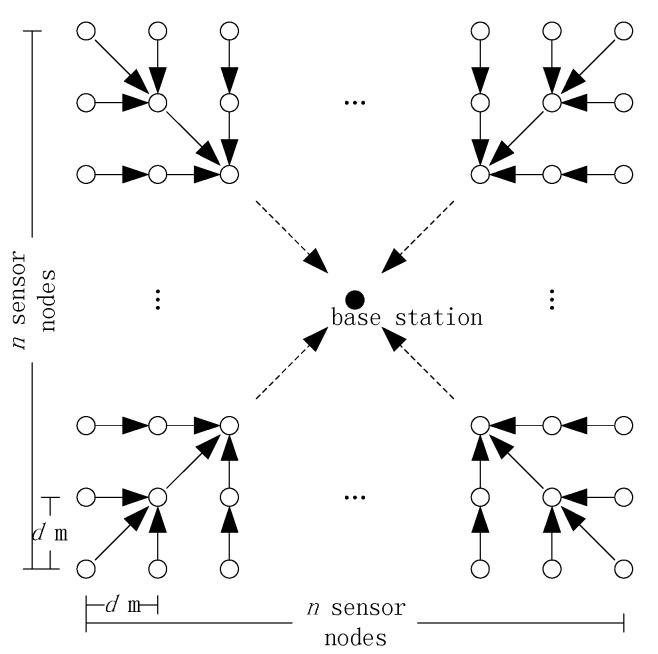
Square-grid topology.

For analytic tractability, consider a square grid consisting of N nodes and the BS located at the center as shown in Figure 6. For FILA and EXTOK, a TAG routing tree [3] is employed by the nodes to communicate with the BS. In the initial phase of constructing the routing tree, the BS needs to broadcast a message asking the nodes to organize a routing tree. In addition, to improve the robust, the tree needs to be updated periodically. For the sake of convenience, the transmission cost of initializing and updating the routing tree is ignored. At the beginning, both FILA and EXTOK need to collect all the readings from the nodes to set filters and the corresponding transmission cost is also ignored.

**Figure 7:**
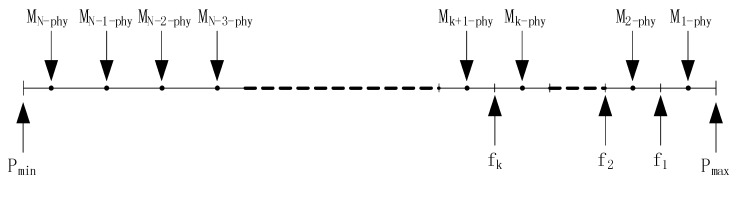
Initial distribution of the readings and filters.

In our analysis, the real value $P_{i}$ of a physical phenomenon for $node_{i}$ is modeled by a normal distribution with parameters $M_{i-phy}$ and $Var_{i-phy}$. The parameters $M_{i-phy},\;i=1,\;2,\ldots ,\;N$ are assumed to be uniformly distributed on the interval [$P_{min}$, $P_{max}$], as shown in upper half of Figure 7. We assume that the initial readings of the sensors equals to the mean values of physical phenomenon, *i.e*.,

(6) $$R_{i}=M_{i-phy}=P_{max}-\frac{P_{max}-P_{min}}{2*N}\times \left(2i-1\right),\;i=1,\;2,\;3,\ldots \;,\;N$$

Based on these readings, the BS calculates the filters based on the method in [1]. A unique filter [$f_{i-1}$, $f_{i}$] is designed for the i-th node in the top-k members and all the other nodes share a common filter $f_{k}$ as shown in lower half of Figure 7. In addition, each node has a normal distributed measurement error $E_{i}$. The reading of i-th node consists of two parts, *i.e*.,

(7) $$R_{i}=P_{i}+E_{i}$$

where $P_{i}$ and $E_{i}$ both are random variable and normal distributed, *i.e*.,

(8) $$P_{i}\sim N\left(M_{i-phy},\;Var_{i-phy}\right)$$

(9) $$E_{i}\sim N\left(M_{i-err},\;Var_{i-err}\right)$$

Based on the properties of the normal distribution, we can get that:

(10) $$R_{i}\sim N\left(M_{i-phy}+M_{i-err},\;Var_{i-phy}+Var_{i-err}\right)$$

When a new query task comes, the reading $R_{i}$ is very likely to change because of two reasons: the changes of $P_{i}$ and affections of the measurement errors $E_{i}$. Therefore, join events, *i.e*., the readings of non- top-k members beyond $f_{k}$, and leave events, *i.e*., the readings of top-k members become lower than $f_{k}$, possibly happen. The probability of join event and leave event for the i-th reading is shown as follows:

(11) $$P_{i}\left(join\right)=P\left(R_{i}>f_{k}\right)=1-F_{i}\left(f_{k}\right),\;i>k$$

(12) $$P_{i}\left(leave\right)=P\left(R_{i}<f_{k}\right)=F_{i}\left(f_{k}\right),\;i<k$$

where $F_{i}\left(w_{k}\right)$ is the cumulative distribution function of $N\left(M_{i-phy}+M_{i-err},\;M_{i-err}+Var_{i-err}\right)$ and it is presented as follows:

(13) $$F_{i}\left(f_{k}\right)=\frac{1}{\sqrt{2\pi \left(Var_{i-phy}+Var_{i-err}\right)}}\int_{-\infty }^{f_{k}}e^{-\frac{{\left(t-\left(M_{i-phy}+M_{i-err}\right)\right)}^{2}}{2\left(Var_{i-phy}+Var_{i-err}\right)}}\text{d}t$$

As illustrated in [1,2], the number of join events and leave events can significantly influence the transmission cost. If |leave|≤|join|, it is not necessary to probe any nodes that are not in the top-k members and the new filters are sent to the relevant nodes rather than all the nodes in the network. However, If |leave|>|join|, to get the top-k readings, all the nodes that are not in the top-k members need to be probed and a new filter is reset for each of them. The probabilities of |leave|≤|join| and |leave|>|join| are shown as follows:

(14) $$P\left(\left|\text{leave}\right|\leq \left|\text{join}\right|\right)=\underset{i=0,\;1,2\ldots ,k}{\sum }P\left(leave=i\right)\underset{i<j<k}{\sum }P\left(join=j\right)$$

(15) $$P\left(\left|leave\right|>\left|join\right|\right)=\underset{i=1,2\ldots ,k}{\sum }P\left(leave=i\right)\underset{j<i}{\sum }P\left(join=j\right)$$

In most practical applications, the parameter k is much less than N which is the size of the network. We can draw this conclusion from actual observations which are described in [1,2]. Therefore, the transmission cost in the condition of |leave|≤|join| is also much less than that in the condition of |leave|>|join|. For the sake of convenience, we focus our attention on the transmission cost in the condition of |leave|>|join| and ignore the transmission cost in the condition of |leave|≤|join|.

In a query, having found that |leave|>|join|, the BS sends a probe message to all the nodes in the network asking them to upload the readings. The transmission cost in this phase is shown as follows:

(16) $$C_{1}=l_{p}*N$$

where $l_{p}$ is the length of a probe message. Having received the probe message, each node transfers its reading to the BS based on a routing tree (e.g., the TAG Tree). Assume that an aggregation technique is employed to reduce the transmission cost and the transmission cost is:

(17) $$C_{2}=k*\left(l_{id}+l_{r}\right)\text{*}N$$

where $l_{id}$ is the length of a node’s ID and $l_{r}$ is the length of a reading. After the BS calculates the top-k readings, a unique filter is generated for each top-k member and a common filter is generated for all the non-top-k members. Then, the BS injects these filters into the network. First, the unique filters are installed by the top-k members. Then, the common filter is broadcasted in the whole network and all the non- top-k members need to install the new common filter. The transmission cost for the filters of top-k members depends on the locations of the members which is random. In average, the transmission cost is $k*\frac{n}{2}*l_{fk}$ in the network as shown in Figure 6. Therefore, the transmission cost of updating the filters is:

(18) $$C_{3}=k*\frac{n}{2}*\left(l_{id}+l_{fk}\right)+N*l_{fc}$$

So the expectation of the total transmission cost for a new query is:

(19) $$C_{total}=P\left(\left|leave\right|>\left|join\right|\right)*\left(C_{1}+C_{2}+C_{3}\right)$$

As in Equation (19), $C_{total}$ is affected by two parts, *i.e*., P(|leave|>|join|) and $\left(C_{1}+C_{2}+C_{3}\right)$. However, $\left(C_{1}+C_{2}+C_{3}\right)$ is constant for a given network. As a result, P(|leave|>|join|) is the most important parameter that affect $C_{total}$ significantly. Based on Equation (15), we can find that P(|leave|>|join|) is mainly affected by the probabilities of a node join or leave the top-k members which are affected by the variance of the readings and the distance between the filter $f_{k}$ and the mean of the reading ($M_{i-phy}+M_{i-err}$). As a result, when the range of the readings [$P_{min}$, $P_{max}$] is constant, N, k and the variance of the readings can significantly affect the transmission cost. What’s more, the dynamics of [$P_{min}$, $P_{max}$] can affect the performance of filter-based top-k query even more significantly.

**Figure 8:**
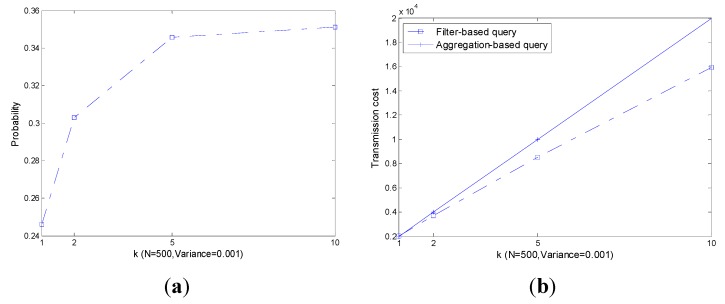
P(|leave|>|join|) and co.rresponding transmission cost with different k. (**a**) P(|leave|>|join|); (**b**) Transmission cost.

**Figure 9:**
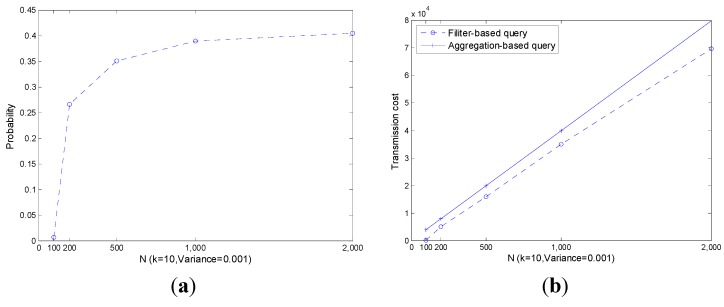
P(|leave|>|join|) and corresponding transmission cost with different N. (**a**) P(|leave|>|join|); (**b**) Transmission cost.

**Figure 10:**
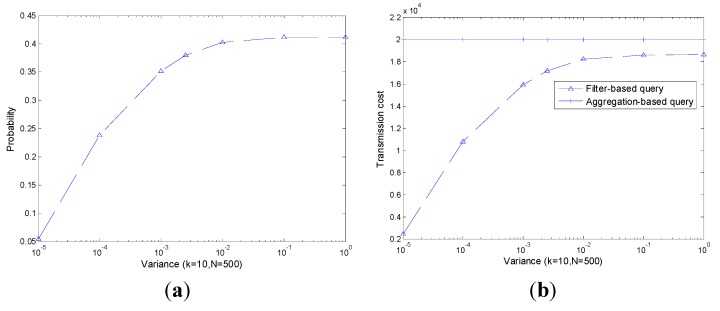
P(|leave|>|join|) and corresponding transmission cost with different $Var_{i-err}+Var_{i-phy\;}\;$(**a**) P(|leave|>|join|); (**b**) Transmission cost.

**Figure 11:**
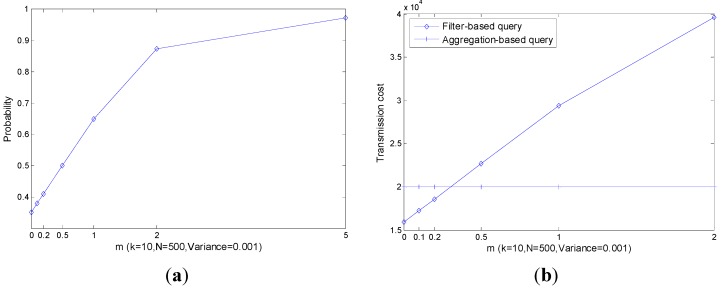
P(|leave|>|join|) and corresponding transmission cost with the decreasing of readings’ range. (**a**) P(|leave|>|join|); (**b**) Transmission cost.

In order to give a visual presentation, we instantiate the parameters and then plot the transmission cost in figures. The parameters are set as in Table 3. First, we fix the range [$P_{min}$, $P_{max}$] of the readings and present the probability of |leave|>|join| and corresponding transmission cost with different parameters including k, N and $Var_{i-err}+Var_{i-phy}$ in Figure 8, Figure 9 and Figure 10. Then, we assume that the reading of $node_{i}$ decreases m times of ${\text{w}}_{\text{i}}$ which is the width of $node_{i}$ ’s filter in a period of query and the simulation results are presented in Figure 11.

**Table 3:** Instantiation of parameters.

Symbol	Value
k	1, 2, 5, 10
*N*	100, 200, 500, 1000, 2000
$l_{id}$, $l_{r}$, $l_{fn}$, $l_{p}$, $f_{i}$	4 bytes
$l_{fk}$	8 bytes
$P_{max}$	30 (constant)
	Decrease by *m* times of *w_i_* in each period of query, m= 0, 0.1, 0.2, 0.5, 1, 2
$P_{min}$	25 (constant)
	Decrease by *m* times of *w_i_* in each period of query, *m =*0, 0.1, 0.2, 0.5, 1, 2
$M_{i-phy}$	$R_{\text{max}}-\frac{R_{\text{max}}-R_{\text{min}}}{\text{n}}\times \left(2\text{i}-1\right)$
$M_{i-err}$	0
$Var_{i-err}+Var_{i-phy}$	$10^{-5}$, $10^{-4}$, $10^{-3}$, $2.5\times 10^{-3}$, $10^{-2}$, $10^{-1}$, 1

As shown in Figure 8a, with the increase of k, the probability of |leave|>|join| also increases especially when k is small. As a result, the transmission cost increases as plotted in Figure 8b. However, the performance of the filter-based query is always slightly better than that of the aggregation-based query.

As shown in Figure 9, like k, with the increase of N, both P(|leave|>|join|) and corresponding transmission cost increase significantly. The performance of the filter-based query is always slightly better than that of the aggregation-based query.

As shown in Figure 10, the transmission cost of an aggregation-based query is independent of the decrease of the readings’ range, *i.e*., the transmission cost is always constant. The transmission cost of a filter-based query increases with the increase of $Var_{i-err}+Var_{i-phy}$, however it outperforms the aggregation-based query all the time.

Though the performance of a filter-based query is affected by k, N and $Var_{i-err}+Var_{i-phy}$, the filter-based query always outperforms an aggregation-based query. Note that, only part of the real transmission cost is presented and the others are ignored. In the following, we present the impact of readings’ range decline on the performance of transmission cost in Figure 11.

Figure 11 shows that the decrease of the readings’ range has a huge impact on P(|leave|>|join|) which is much bigger than the other parameters discussed previously. As shown in Figure 8, Figure 9 and Figure 10, the limit value of P(|leave|>|join|) is about 0.5 with the increase of the parameters. However, in Figure 11, the limit value of P(|leave|>|join|) is 1. As a result, when m≥0.5, we can find in Figure 11b that the transmission cost of a filter-based query is much larger than that of the aggregation-based query. In this situation, filters in the network are useless and a more efficient top-k query approach is needed.

In conclusion, filter-based top-k query approaches are very sensitive to the size of the networks, dynamics of the sensors’ readings and decline of the whole range of the readings. In some situations, the filters can’t improve the performance of query approaches significantly. What’s more, in some certain situations, the filters are useless and even become the burden of approaches. Therefore, it is very meaningful to design a more efficient top-k query approach.

### 5.2. Simulation Setup

**Figure 12:**
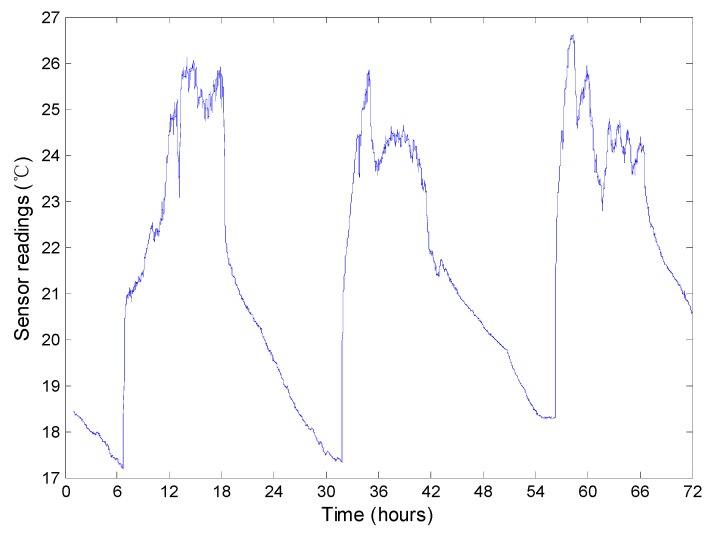
The temperature readings of the No.1 node from March 1st to 3rd.

In our simulation, 500 homogeneous sensor nodes are randomly scattered in a 200 m × 200 m region. For each simulation, to reduce the randomness of the simulation result, we do the same experiment for 10 times and present the average result. The temperatures contained in Intel Berkeley dataset [19] is used to simulate the readings of the sensor nodes. Millions of pieces of recordings, including temperature, humidity, light and voltage, comprise the dataset generated by 54 sensor nodes deployed in the Intel Berkeley Research lab. Figure 12 presents the temperature readings of the No. 1 node from March 1st to 3rd. For each day, we find that the temperatures increase from about 7 o’clock to 14 o’clock, fluctuate from about 14 o’clock to 18 o’clock and decrease from about 18 o’clock to 7 o’clock in the next day. As discussed previously in Section 5.1, the decrease of the readings has a strong effect on the performance of the approaches. Therefore, we can perform an overall evaluation on the top-k query approaches using the dynamics of the readings.

As the number of sensor nodes in the dataset is 54 and it is much smaller than that of our network, we need to design a dispatcher to dispatch the readings to 500 nodes considering the spatial correlation of sensor readings. First, we divide the 500 nodes into five clusters based on algorithm 1 and, for each cluster, select a representation located in the center.

Then, the readings of five nodes in the Intel Berkeley dataset are randomly selected and we extract the readings of each node in a random day. Then the readings of the five nodes are dispatched to the five clusters in our network respectively, *i.e*., every node’s readings in a day are dispatched to a cluster. In Intel Berkeley dataset, one node generates about 2000 readings in a day and the largest cluster in our network has about 150 nodes. As a result, it is enough that every node in our network can receive 10 readings and, therefore, each experiment can perform the query 10 rounds. Considering the temporal correlation, first, the readings for a node in Intel Berkeley dataset in a day are divided into 10 subsets based the time sequence. Then the number of the nodes in a cluster in our network is calculated and denoted by $N_{i}$. In each subset, we randomly select $N_{i}$ readings. Intuitively, considering the spatial correlations between the readings, for each cluster, the representation has the highest reading and the other nodes’ readings decrease with the increase of the distance to the representation.

**Figure 13:**
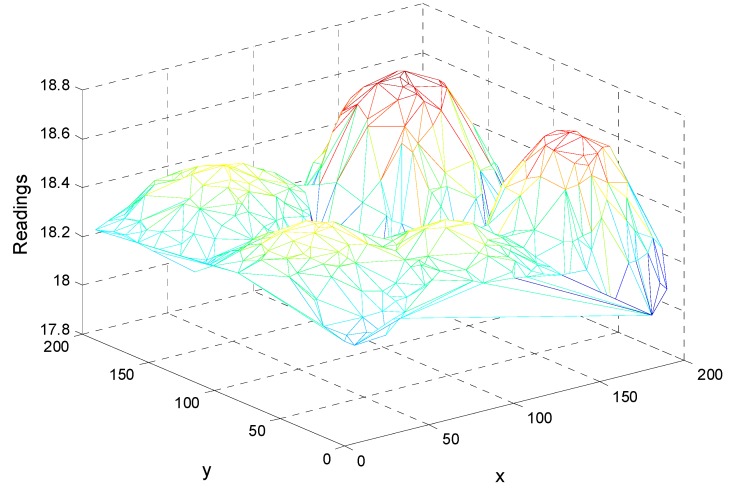
An example of the readings in a query round.

**Figure 14:**
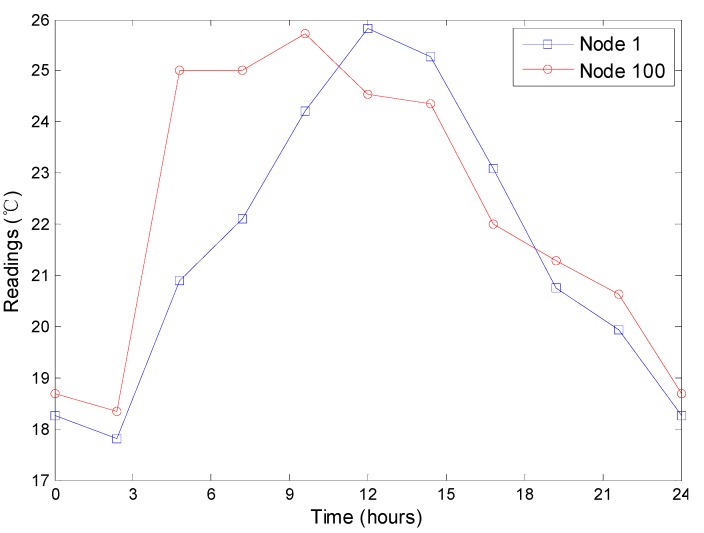
An example of the readings of a node in a simulation.

An example of the readings for a round of query is shown in Figure 13. There are five extreme values in the overall network and the spatial correlation is also presented. In our simulation, each sensor node has ten readings in chronological order and each reading corresponding to one query round. The ten readings fluctuate as shown in Figure 14, which is similar to a period of the readings shown in Figure 12 to some extent. Note that the ten rounds rather than one round of top-k query comprise an experiment.

We compare our approaches with TAG, FILA and EXTOK in terms of transmission cost, query accuracy, energy cost and network lifetime. In our simulation, a sensor node identifier and reading both take 4 bytes.

### 5.3. Transmission Cost and Query Accuracy

**Figure 15:**
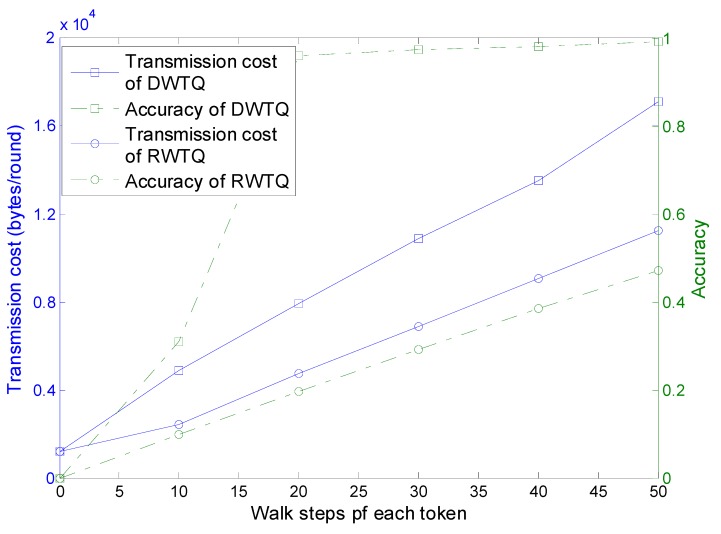
Transmission cost and query accuracy.

For TAG and EXROK, the query results are the exact top-k readings in the networks, however, the query results of FILA have deviations which are affected by the properties of the network and the queries. The results of RWTQ and DWTQ also can’t be guaranteed to be the exact top-k readings. We define the query accuracy ρ as follows:

(20) $$\rho =\frac{\left|Results\cap^{\text{​}}Top_{k}\right|}{\left|Top_{k}\right|}$$

where Results is the query results of the base station and $Top_{k}$ is the real top-k readings in the network. In this part, five tokens are injected into the network and we set k=l=10, m=4, t=0.7, ang=90°. For the different parameter T which controls the walk distance of a token, the transmission cost and query accuracy of RWTQ and DWTQ are significantly different. The simulation results are presented in Figure 15.

As the walk steps increase, the transmission cost and query accuracy of both DWTQ and RWTQ increase significantly. As shown in Section 3 and Section 4, the information contained in the tokens of DWTQ is larger than that of RWTQ, therefore, the transmission cost of DWTQ is always larger than that of RWTQ when their walk steps are equal. In addition, for the same walk steps, the query accuracy of DWTQ is much higher than that of RWTQ. However, we focus on the relationship between the transmission cost and the query accuracy. We can find in Figure 15 that when the transmission cost is similar, then the accuracy of DWTQ is much higher than that of RWTQ. As an example, when DWTQ takes 1600 bytes in a round, the average accuracy is about 0.98 and the accuracy of RWTQ is smaller than 0.4. In conclusion, DWTQ outperforms RWTQ in transmission cost when the accuracy is set to be a constant in our simulation environment. In the following simulations, we use DWTQ to compare with the existing approaches.

We now compare the transmission cost between DWTQ, TAG, FILA and EXTOK. In this simulation, each token walks 25 steps in the network. Different with traditional simulation, each experiment contains ten rounds of queries in a day in chronological order. The initial transmission cost for constructing routing trees and installing filters in TAG, FILA and EXTOK are ignored.

**Figure 16:**
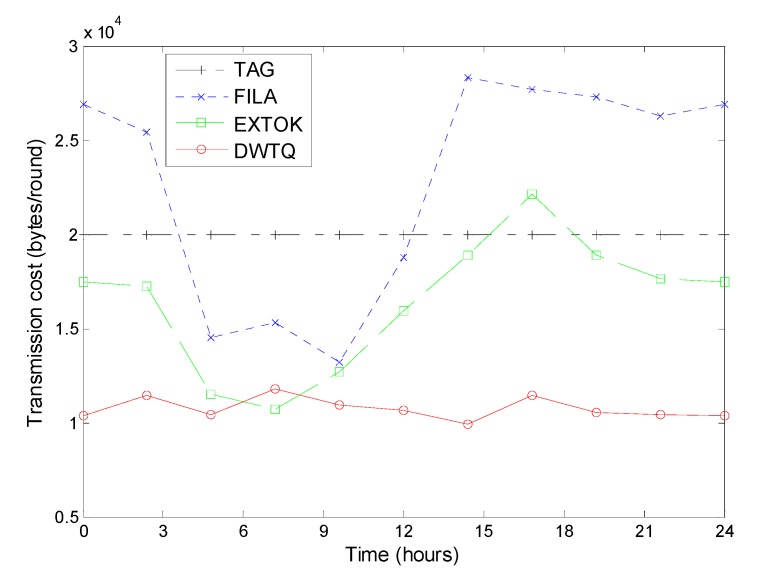
Transmission cost versus different time.

As shown in Figure 16, at any time, the transmission cost of TAG and DWTQ is always relatively constant; on the contrary, the performances of FILA and EXTOK are very sensitive to the fluctuation of the temperature. When the temperature increases, the transmission costs of FILA and EXTOK are much smaller than that of TAG; when the temperature decreases, TAG outperforms FILA and EXTOK in transmission cost.

In most cases, the transmission cost of DWTQ is smaller than that of three other approaches. The reason is that the transmission cost of DWTQ is independent with the fluctuation of the readings and DWTQ makes full use of the spatial correlations between the readings. We should note that DWTQ trade query accuracy for communication overhead though the decreasing of the accuracy is very small in most cases.

### 5.4. Energy Cost

**Figure 17:**
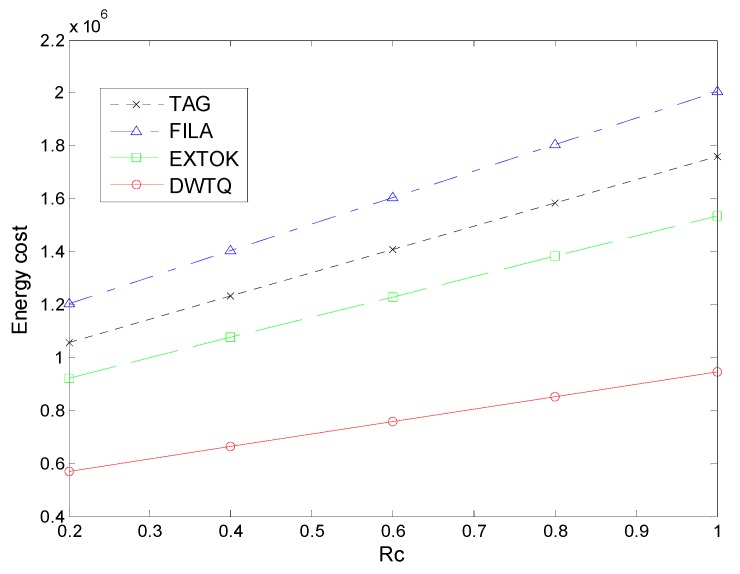
Total energy cost for a node in a day with different $R_{c}$.

As in [2], to escape the technology affection, we assume that the unit of energy required for transmission of a single bit, $E_{tx}$, and we use a parameter, $R_{c}$, to link transmission and reception cost, $E_{rx}$, *via* $R_{c}=\frac{E_{rx}}{E_{tx}}$. In our simulation, $R_{c}$ is assigned values from the set {0.2, 0.4, 0.6, 0.8, 1.0} and the other parameters is the same to that in Section 5.3. The simulation result is shown in Figure 17.

As the cost of reception increases, the overall energy increases for all the approaches and we can find that the increase of DWTQ is the slowest.

### 5.5. Network Lifetime

**Figure 18:**
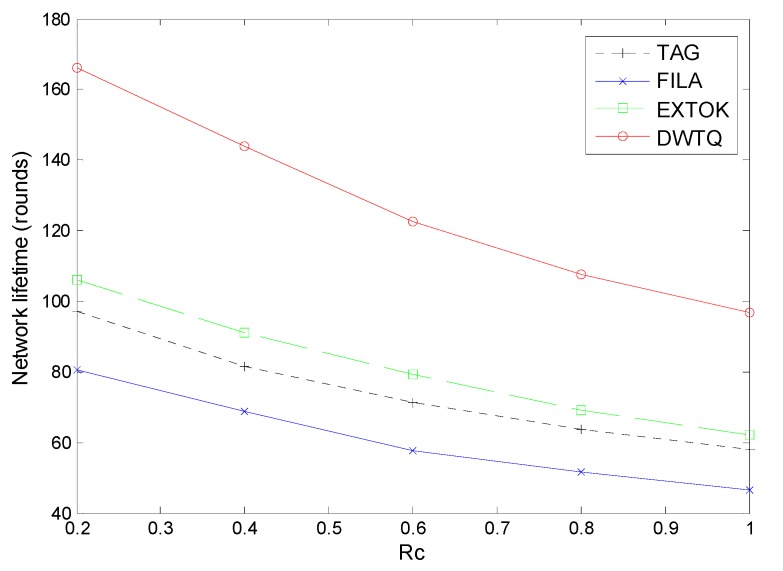
Network lifetime with different $R_{c}$.

At last, we evaluate the performance with respect to the network lifetime which is defined as the number of rounds before the first node runs out of its energy. The initial energy for each node is set to $10^{8}$ energy units and the network lifetime with different $R_{c}$ is presented in Figure 18.

As the $R_{c}$ increases, the network lifetime of all the approaches decreases. However, the simulation results reveal that the DWTQ significantly prolongs the lifetime compared with the three other approaches. In particular, when $R_{c}=0.6$, DWTQ can be operated about 120 rounds, *i.e*., 12 days, which is about 1.5 times the duration of EXTOK and 2 times that of TAG.

### 5.6. Concluding Discussion of DWTQ

Through a series of simulations, we can find that DWTQ outperforms TAG, FILA and EXTOK in transmission cost, energy cost and network lifetime. This can be explained by the fact that DWTQ makes full use of the spatial correlation of the readings and its performance is robust to the decline of the overall range of the readings. However, DWTQ can’t guarantee the query results are exactly the top-k readings in the network. This is the weakness of DWTQ compared with TAG and EXTOK. Therefore, the users have to choose a proper top-k query approach for different conditions. Obviously, if the users can tolerate some random errors, DWTQ would be the best choice.

## 6. Conclusions

In WSNs, most of the top-k query approaches employ aggregation or filtration techniques to reduce the transmission cost and save network energy. Often, the filter-based approaches outperform the aggregation-based approach, however, they are too sensitive to the parameters, especially the overall descent of the readings. In addition, the approach based either on aggregation or filtering technique doesn’t consider the spatial correlations of the readings. Leveraging the random and directed walk techniques, two novel top-k query approaches, RWTQ and DWTQ, are proposed. A series of simulations presented in Section 5.2 illustrate that the proposed paradigm DWTQ is very robust against the dynamics of the sensors’ readings and decline of the whole range of the readings. In addition, we find that aggregation-based approaches are very general methods and they have a large traffic; filter-based approaches on the other hand are too sensitive to the temporal characteristics of the readings and have a small traffic when the readings are stable to some degree; DWTQ is very sensitive to the spatial characteristics of the readings and RWTQ is general has a low accuracy. In applications of WSNs, the spatial correlation is very common and, in this condition, DWTQ outperforms other approaches in transmission cost and lifetime of the networks.

As future work, we plan to explore the following topics: (1) whether we can further improve the performance of the proposed approaches based on employing sophisticated optimization methods or not; (2) whether we can reduce the time complexity for the nodes when deciding the walking direction or not; (3) whether we can design a self-adjusting filter to defend against the dynamics of the physical phenomena based on the temporal correlations of the readings or not.
